# A content analysis of nature photographs taken by Lebanese rural youth

**DOI:** 10.1371/journal.pone.0177079

**Published:** 2017-05-10

**Authors:** Miriam Mattouk, Salma N. Talhouk

**Affiliations:** 1 Nature conservation center, American University of Beirut, Beirut, Lebanon; 2 Department of Landscape Design and Ecosystem Management, Faculty of Agricultural and Food Sciences, American University of Beirut, Beirut, Lebanon; TNO, NETHERLANDS

## Abstract

‘Living in Harmony with Nature’ is a 2050 vision put forth by the Convention on Biological Diversity (CBD) which takes into consideration culture and locality of perceptions of nature and aspirations for its use. Considering that 54% of the world population lives in cities, where nature has been decimated, the role of rural communities, living within or in proximity of natural and semi natural areas, will effectively influence the fate of the ‘remaining’ nature while they engage in local development. Reconciling between growth and development on the one hand, and nature conservation on the other, necessitates an understanding of how rural communities, especially the youth, imprint their own ideas on landscapes and develop ownership over natural spaces. In order to extend the understanding of how harmony with nature is perceived in different parts of the world, this paper presents the findings of primary research involving a group of young people who live in rural areas in Lebanon, a country in the Arab Middle East. Participatory research based on photovoice methodology was conducted with 77 young people aged 7–16 and residing in five rural villages located in different parts of Lebanon. Photographs taken by participants indicated that for many, nature was not perceived literally i.e. woodlands, forests, plants, animals, etc…. Instead, the participants saw nature as part of agriculture and local culture. Nature was also seen as symbolic expression of the participants’ inner state of mind. Narratives written to explain the photographs shed light on the perception of harmony with nature which focused on positive family experiences and relayed personal emotions, abstract, and holistic yet functional view of nature. Another recurring theme that emerged from photographs and narratives was the role of family members, in particular grandparents, as having a strong influence on the positive perceptions of nature.

## Introduction

‘Living in Harmony with Nature’ is the 2050 vision of the Convention on Biological Diversity (CBD). This concept, introduced and adopted as a resolution in 2009, is the basis of a global strategy that considers as fundamental the interconnection between humanity and nature (www.harmonywithnatureun.org/chronology.html; accessed June 8, 2016). The vision is comprehensive as the word harmony has multiple meanings that reflect possible relations between humans and the world they inhabit. Consensus, consistency, cooperation, friendship, peace, rapport, tranquility are all synonyms to harmony. On the other hand, antonyms of the word shed light on problems faced in the absence of harmony; disagreement, discord, dislike, hatred, incompatibility, fighting, imbalance, and disproportion. The word harmony most likely has also different meanings in different languages; For instance, in Arabic, harmony is translated in CBD texts as ‘insijam’. This Arabic term is used to indicate harmony in music, but also to reflect a proactive role taken by a person to overcome internal conflicts to reach a state of harmony with him/herself. It also points to a person in complete alignment with others; he/she ‘Insajama’ with people. From this perspective, harmony with nature is not only influenced by external factors but also by factors internal to individuals and communities. Accordingly, from the perspective of an Arabic speaker engaged in conservation research, ‘living in harmony with nature’ is a vision that calls for consideration of discords at individual and communal levels, in order to achieve personal/social ‘insijam’ (or harmony) as a means to reach harmony with nature. Such an interpretation of the vision is relevant to many Arab countries where large numbers of ordinary citizens live in politically unstable environments and military conflict areas. In this respect, the reality that cannot be overlooked is that local communities struggle to meet basic human needs and this takes precedence over concerns to one’s natural heritage which is prone to destruction by war, abandonment, encroachment and plunder (www.almaany.com/ar/dict/ar-ar/; accessed June 1, 2016); www.harmonywithnatureun.org/chronology.html) accessed May 17 2016.

Considering that 54% of the world population lives in cities with limited access to ‘nature’ (www.un.org/en/development/desa/news/population/ world-urbanization-prospects-2014.html), indigenous cultures and rural communities will most likely effectively influence the fate of nature because currently they are the immediate beneficiaries and potential guardians of natural areas. Decisions related to future lifestyles, economic activities and urban development taken by these communities living outside cities, where nature has already been lost in a large measure, will determine whether there will be further destruction or attempts at reconciliation. Hence local understanding of the perception of nature will allow for better alignment between local and global visions of harmony with nature.

In order to extend the understanding of how harmony with nature is perceived in different parts of the world, this paper presents the findings of primary research involving a group of young people who live in rural areas in Lebanon, a country in the Arab Middle East. The study illustrates the importance of addressing children’s own understandings of nature and provides an opportunity to present children as key agents of change for nature conservation (www.cbd.int/ibd/2008/youth/action/ accessed September 9, 2015).

Engagement in nature at an early age allows youth to materially and emotionally imprint their own ideas on landscapes and develop symbolic ownership over space. Reporting on their study that explored the youth experience of nature as part of their mountain biking activities, the authors show how young people establish connections to the land by negotiating access restrictions and shaping meaningful spaces for themselves [1]. By developing intimate connections with spaces often unnoticed by the adult community, young people differentiate their relations with nature from those developed by adults.

Nevertheless, appreciation of nature is mostly culture dependent as shown in a study conducted with Kenyan children where culture was found to be the ‘raw material out of which children fashion and shape their environmental meaning’[2].

Located on the Eastern shore of the Mediterranean sea Lebanon is a small predominantly mountainous country (10,452 km^2^) consisting of a narrow coast line and two mountain chains (up to 3087m), running parallel to the Mediterranean coast and separated by a high plateau. The Lebanese coast line is severely damaged by urban expansion that accommodates 2/3 of the population estimated at 4 Million [3]. In contrast, population density in Lebanon’s mountains is relatively low; towns and villages are part of a mosaic that consists of semi-natural mountain ecosystems, including forests, woodlands, and open spaces, agricultural lands, and cultural landmarks all integrated to form the Lebanese natural and cultural landscape. This situation unfortunately is in the process of change. With increased population density, towns and villages are expanding away from traditional Mediterranean farming lifestyles that rely on multifunctional uses of lands. Instead, local village and town residents are adopting urban lifestyles where growth and livelihoods develop independently of sustainable management of natural resources. This change coupled with an increase in built areas will undoubtedly influence the fate of remaining nature relics, and this in turn will determine the fate of the natural heritage of the country as a whole.

## Research methods

### Case study locations

**Fig 1:**
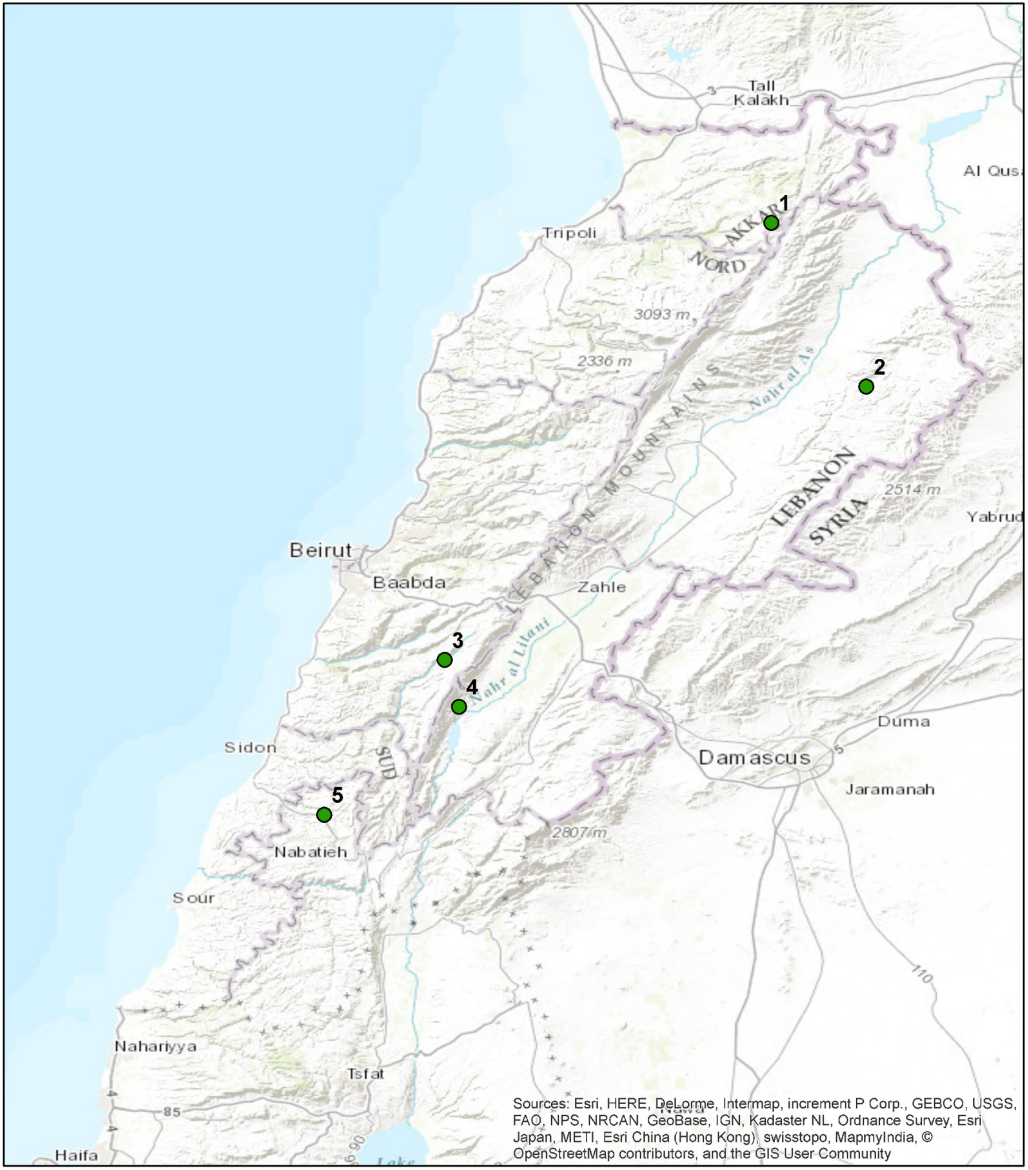
Location of case study villages in Lebanon. 1, Michmich (S4), 2, Arsal (S1), 3, Barouk (S3), 4, Ain Zebdeh (S2) 5, Deir Zahrani (S5).

This paper draws upon research with young people residing in five rural villages located in the north, center, and southern parts of Lebanon (Fig 1). The case study villages are located in rural settings which consist of a mosaic of natural (woodlands), agricultural (terraces) and cultural (archeological sites and traditional neighborhoods) landscapes covering more than 90% of the political boundaries of these villages with the remaining consisting of built areas. The built areas consist of two to three floor buildings expanding out of the historical old village centers. The villages and communities are diverse; they are located at altitudes ranging from 400 m asl to 2000 m asl, population sizes vary from 3500 to 35000, and each village represents different religious affiliations. The selection of these villages was based on standing relations between the University researchers (Nature Conservation Center at the American University of Beirut (AUB-NCC) and the community where reforestation programs and outreach activities are conducted [4].

### Photovoice

The qualitative research methodology employed is based on ‘photovoice’ described as allowing people to identify, represent and enhance their community through photography [5,6]. Photovoice was further defined as having three goals: 1) enable people to record and reflect their community’s strengths and concerns; 2) promote critical dialogue and knowledge about personal and community issues; and 3) reach policy makers [7]. Wesley (2011) [8] indicated that photovoice addresses three core criteria: community-centered control, knowledge production, and outcome-oriented results. The first Photovoice project was undertaken with women living in rural China; photographs taken by women were used to discuss concerns about health and living conditions in their community [5,9]. One photovoice approach, designed by the Mwelu Foundation, emphasized the development of photography, documentary video and journalism skills in participating youth of the Mathare community in order to communicate their problems to a wider audience [10]. Another study focused less on photographic technique and more on the critical discourse and representation of participants’ life experiences to others [6,11,12]. Bellin (2006) [13] explored photo-elicitation with farmers, giving them a means to express the complexities of being a farmer through the use of a camera.

Some authors reported limitations of photovoice with complications arising as a result of the flexibility granted to communities in determining the individuals that participate, and in proposing the extent and duration of participation [14]. In our approach we adopted existing local mechanisms by working through local schools, school administrators, teachers, students, and parents. Others reported incidences where photovoice failed because communities were skeptical about the benefits of the approach, were unwilling to participate, disliked photography, were unreliable about their photographic input, and in some instances even damaged the cameras provided to them [15,16]. We experienced the opposite response from the youth; to keep the participants engaged and committed to the whole process, we adopted a short time limiting workshop sessions to two, an introductory and an analysis workshop, and giving the participants seven days to take photos. This decision resulted in zero dropout occurrences, as compared to the higher number of dropouts in similar projects. It is possible that the observed success was due to the fact that students in rural communities are rarely exposed to after-school activities; they were eager and engaged in all aspects of the process and openly shared their perception of nature with the team.

Showing all the photographs and quotes taken by the participants goes beyond the scope of this article. These have been archived digitally in ‘Word’ documents that do not disclose names to protect participants’ confidentiality, but it is important to highlight the fact that most participants were extremely open and welcoming to the workshops and presented surprisingly attractive and stimulating photographs and narratives. The youth engagement in the participatory process could be illustrated in various anecdotes and informalities that established a positive relation between instructors and participants. In most cases the outdoor practice sessions ended with the youth ‘dragging’ the instructors into a fun village tour. In all cases the instructors were warmly received and the adopted informality in the workshop sessions allowed other children from the village drop in during the session and participate at will during the day. In one village, some students hid in the trunk of the instructor’s car and had a laugh getting out. In another village, the youth loved the photography aspect of the workshop but they showed even more interest in the prospect of writing narratives to express their ideas.

### Participants and ethical considerations

The study took ethical considerations and consulted with the University IRB. Invitation of the participants was achieved primarily through direct contact with local public school administrators. Initial visits were made to local schools and meetings were held with administrators and teachers to explain the project and its objective. Based on the advice of the schools, it was agreed to plan the activity similar to any other optional afterschool activity organized by the school. The team gave a presentation to the students that were gathered by the school for a presentation of the project. School representatives were provided with a call for participation letter explaining the project, the workshops, the activities, and the outcome. These were distributed to the students and their parents. Following the introductory meeting with students, and the dissemination of the call for participation letter, the school representatives collected the contact information of students who showed interest in participating and who received their parents’ oral consent. The workshop schedule was arranged with the school representatives after they consulted with the students who expressed interest to participate and their parents on a most suitable time schedule.

A total of 77 young people between the ages of 7 and 16, registered in the workshops which were advertised through the local schools. In each village, the participants attended a two-day workshop which was held as an after-school activity in the local school and which included field, artistic and team building activities revolving around responsibility and follow-up, creativity, self-expression, and tangible outcomes. Upon the workshop’s completion, the participants were handed digital cameras and notebooks to record and represent, through photographs and narratives, their perceptions of nature.

### Workshops

The workshops were developed to help conduct research with children and not on children.

For every workshop the following supplies were used: Flipchart, markers, pencils/pens, note cards, scissors, staplers, tape, and refreshments. Each participant received a package containing a point-and-shoot camera (to be returned upon completion of the workshop), pencils, a notebook, an extra set of batteries, and a printed manual containing introductory information about theory and practice of photography covered during the workshop.

Participants were then introduced to the project and to each other through an ice-breaking activity; a large white cardboard was placed on the wall and participants were asked to take turns writing a nickname that best describes them and to explain why they feel the name applies to them. The participants were then given post-it notes on which they were asked to write one word describing nature (i.e. birds, trees, leaves, etc.) and stick it somewhere on the cardboard.

The instructors then described the contents of the package received by each participant and they explained how photovoice has been used in other countries. The purpose was to increase their understanding of the project objective and the importance of their contribution.

The participants were then given an introductory photography training session. They learned how to use the cameras, how to take a photograph, and describe it through a narrative. The participants then moved outdoors, on a camera training session, to practice their newly learned skills under the guidance of a professional photographer who gave helpful one-to-one feedback. At the completion of the workshop, they were given assignments to take as many photographs as they like then select three photographs and quotes on nature. We explained to participants the freedom through which they can think about nature: how do you see nature, or who/what made you care for it, or what would you like to keep for future generations.

The second workshop started with a group discussion about the week’s photography assignment. Each participant then presented and discussed photographs and narratives that they selected out of the many photographs taken during the week. The instructors encouraged questions and facilitated discussions of photographs which were projected on a screen. The pictures were used to stimulate group discussions about environmental, personal and community issues. With every photograph, the participant read the quote intended for it out loud and talked about it.

### Analyzing photographs

Thematic analysis of photographs first involved identifying and grouping images according to different themes (Table 1). These groupings were conducted independently by the author and the co-author. Once completed, the author and co-author discussed the findings, clustered the photographs and named the themes. Photographs were organized into three categories namely: landscape, focus, and symbolic images. The Landscape category included photographs that represented visible features of an area of land such as mountains, water bodies such as rivers, as well as manmade landscapes such as farms and orchards, and cultural sceneries such as villages and neighborhoods. The focus category included photographs that focused on an element be it an organism such as a plant or animal, a tool, or a natural element such as the sun. The symbolic category included abstract or literal images that represent, stand for, or point to something else. There may be a variety of ways to interpret symbolic shots, as each person can understand it differently. That is why the narratives accompanying the photographs helped a great deal in coming to a closer understanding of their message. The landscape and focus categories were further divided into three themes: natural, agricultural, and cultural. After the categories were agreed upon and defined, the authors independently assigned photographs to the categories.

**Table 1:** Themes and number of photographs taken under each them by Lebanese rural youth, ages of 7 and 16, to illustrate how they see nature, who or what influenced them to care for nature, and what they would like to keep for future generations (n = 77).

School	Number of participants	Nature	Agriculture	Culture	Symbolic
**S1**	13	0	1	5	7
**S2**	14	1	6	6	1
**S3**	16	3	8	3	2
**S4**	11	5	5	1	0
**S5**	18	6	5	5	2

### Analyzing narratives

Thematic analysis of texts involved the identification and grouping of key words or phrases throughout the data set. These groupings were done independently by author and co-author. Once completed, the author and co-author discussed the findings and named the themes. The texts were read independently of the photographs and the authors agreed on two main categories. Quotes which fell under the ‘other’ category and included mentions of the family, future generation, and knowledge related facts, and the ‘self’ category which consisted of personal reflections, thoughts, and emotions related to nature.

## Results

### Photographs

**Fig 2:**
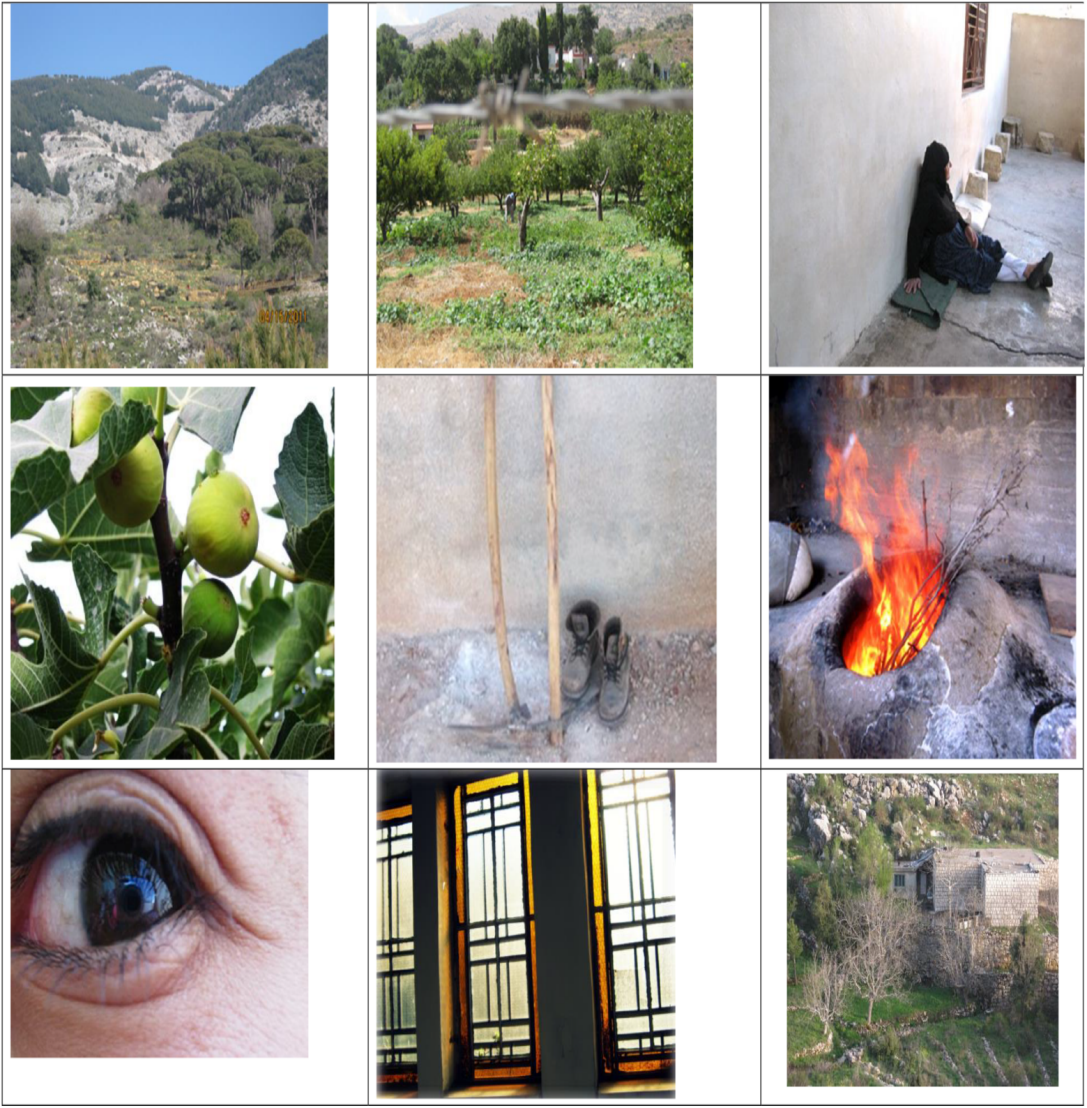
Sample photographs taken by Lebanese rural youth to express their perception of nature.

The photographs taken by participants reflected their visual perception of nature and were very diverse (Fig 2). Some participants took photographs of natural landscapes showing features of the land, including the physical elements of landforms such as mountains, hills, water bodies such as rivers, lakes, ponds and the sea, and living elements including rocky outcrops, woodlands and forests. Some focused on elements of nature such as the sun, a close up shot of a water feature, a plant, or an organism. Others saw nature through orchards, fruits, and agricultural tools including field boots. For some, nature was cultural reflection and included traditional stone houses, traditional furnaces for bread making, and very often family members including grandparents, parents, and the extended family. Finally, some participants saw nature as an inspiration and an opportunity for abstract reflections.

**Fig 3:**
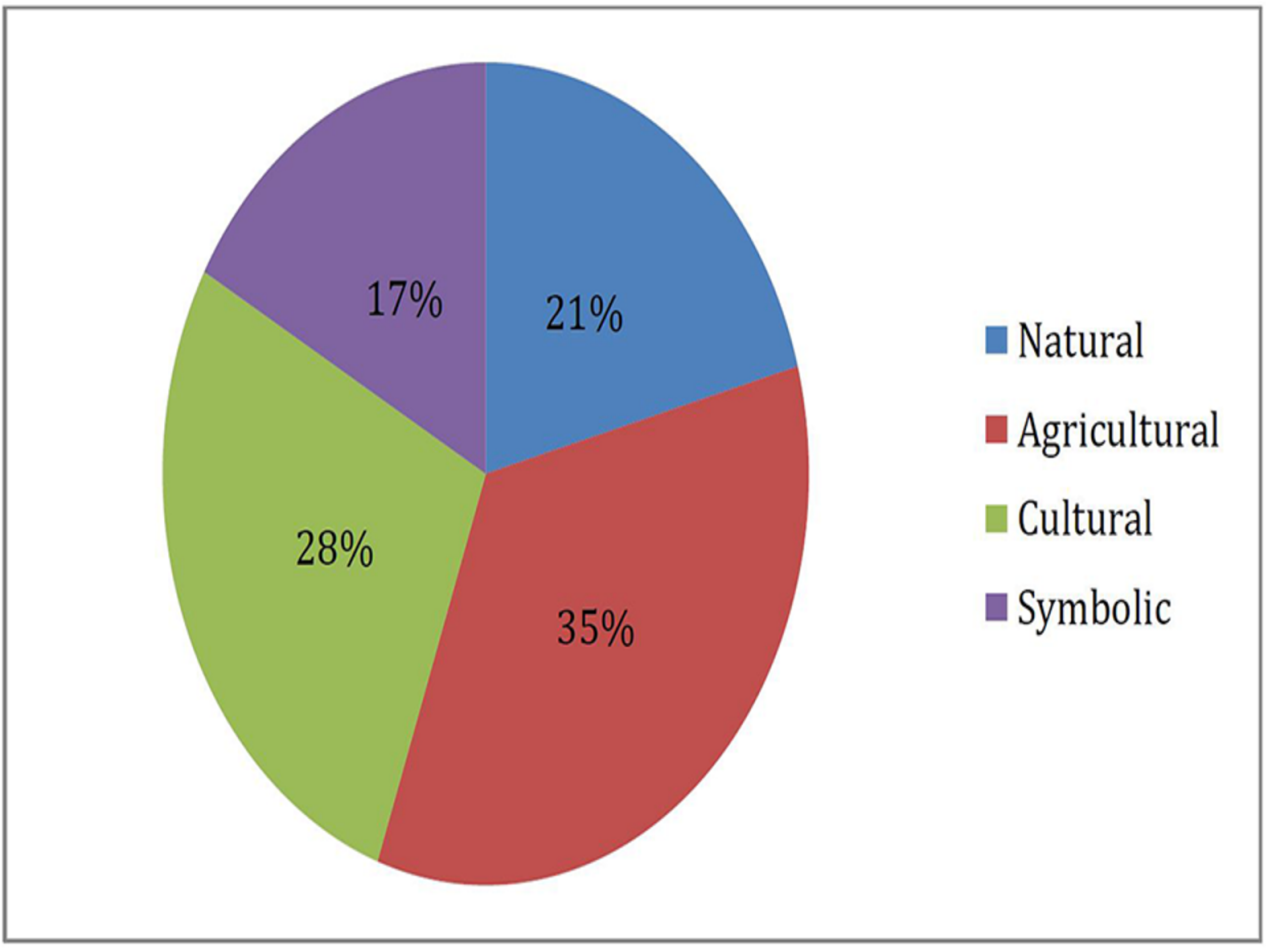
Pie chart representing relative frequency at which Lebanese rural youth (aged 7–16) took photographs of nature falling under different themes (n = 77).

For many of the participants, photographic shots of nature were not literal i.e. woodlands, forests, plants, animals, etc…. Instead the photographs taken by the youth revealed the intricate ways with which they chose to represent nature (Table 1 and Fig 3). Only one fourth of participants opted for the ‘literal’ perception of nature (15 photographs, 21%). The remaining saw nature ‘indirectly’ through agricultural activities (25 photographs, 35%), and in their daily cultural lives (20 photographs, 28%). Nature was also conceived symbolically (12 photographs, 17%). There may be a variety of ways to interpret symbolic shots, as each person can understand it differently. That is why the narratives offered by the participants were essential to understand their perception of nature.

### Narratives

Narratives written to explain the photographs were equally intriguing and shed light on the perception of harmony with nature as expressed by the participating youth. By going through these narratives we endeavored to reach some part of the children’s perceptions of nature to draw attention on the priorities and issues highlighted by them and which would remain with them as they grow up.

**Fig 4:**
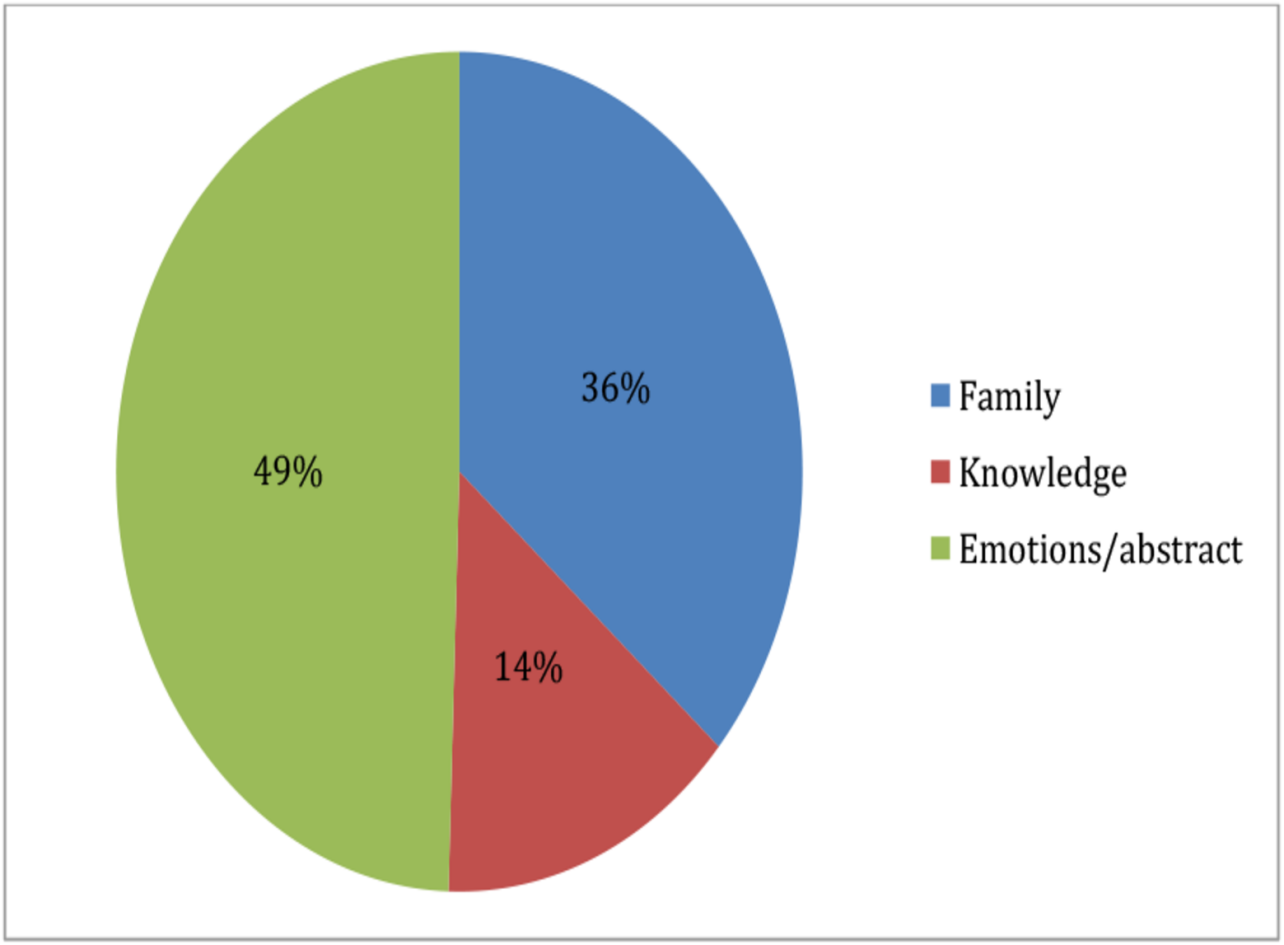
Pie chart representing relative frequency at which Lebanese rural youth (aged 7–16) narrated photographs of nature using different theme topics (n = 69).

When asked to write about their perception of nature, the youth chose to focus on positive family experiences (25 quotes, 36%) as shown below (Fig 4).

‘Nature gave me my grandmother because it kept her healthy’

‘I am grateful for my dad who made me love and care for nature. This love was developed because I used to go with my dad to the bush to mulch and care for trees and cherries. He used to return wearing his planting boots’

‘Nature for me is these simple things because they are the tools we use to beautify it. Through this we have trees and flowers. The shoes are those that I use to work’…

The youth relayed personal emotions, abstract, and holistic yet functional view of nature (34 quotes, 25%)

‘Nature is our soul. With its beauty, I can love it and make everything remain beautiful. It is full of secrets and miracles’

‘Nature is half of the ‘man’, if it is present we will be present, and if we care for it, it will care of us’

‘Nature is where I go out and play, nature is outdoors’

Some preferred to present nature by sharing biological facts (10 quotes, 14%).

I would like my generation to care for as many trees as possible so that we can increase them instead of decrease them for the generation to come…

‘When the kids grow up, the trees will be taller and the area that is empty will be covered with trees’

The narratives suggest that only a small number of narratives explained nature based on learned facts while all remaining were related to positive emotions derived from family, from personal experiences, and from creative abstract expressions of relations with nature (Table 2).

**Table 2:** Themes and number of narratives produced under each theme by Lebanese rural youth, ages of 7 and 16, to illustrate how they see nature, who or what influenced them to care for nature, and what they would like to keep for future generations (n = 69).

School	Number of participants	Family	Knowledge	Emotions/abstract
**S1**	11	1	4	6
**S2**	14	13	0	1
**S3**	16	2	2	12
**S4**	10	5	2	3
**S5**	18	4	2	12

## Discussion

The photographs taken in this study revealed that the youths’ contexts influenced their perception. They used their experiences to visually share their understanding of nature and relay their positive relation with it. In more than 50% of the cases, the youths’ ideas related to services provided by nature such as agricultural and cultural. Consequently these findings shed light on the importance of Strategic Goal D as an entry point for addressing this and the remaining strategic goals of the strategic plan for biodiversity 2011–2020 (Goal D: enhance benefits to all from biodiversity and ecosystem services; www.cbd.int/sp/default.shtml). These findings also shed light on the fact that perception of nature is diverse and efforts to engage children in biodiversity should go beyond formal learning by educators; they should include the home and the informal outdoors, and focus on local biodiversity. The importance of knowledge of local biodiversity is essential to ensure a harmonious relation with nature. A study by Ballouard et al. (2011) [17] called on media and schools to engage children in developing favorable attitudes toward local biodiversity. Their studies revealed that children in European and other case study countries are influenced by internet and other media in that they are more prone to recognize and protect exotic charismatic animal species than their own local species.

Nature as agriculture was a recurring theme in group discussions, photographs, and quotes. Agriculture as nature was illustrated by the youths who felt more comfortable and compelled to perceive nature as gardens, gardening, and orchards, rather than forests, woodlands, and wildlife.

Agriculture in Lebanon and other Mediterranean countries corresponds to the well-organized agro-pastoral rural living that sustained itself for thousands of years and led to a dynamic coexistence of human and natural living systems, providing stability, while fostering diversity and productivity [18]. The domestication of plant and animal species, which first occurred in the eastern Mediterranean area contributed to the increase of certain components of biodiversity while cultural practices have maintained intermediate levels of disturbance promoting biological diversity and keeping ecosystems robust and resilient [19].

The cultural perception of nature in its agricultural context pin points at local levels to informed interventions that may help establish microhabitats contributing to local scale heterogeneity. Furthermore, like elsewhere in the Mediterranean Basin, the ecogeography of the country is such that unproductive pieces of land, hard to manipulate, such as cliffs, mountain slopes, ponds, swamps, or rocky-bare soils will need to be considered for uses alternative to agriculture [20].

By building on expressed local priority, i.e. agriculture, the nature conservation strategy would not be based on conventional messages, information, and action that put biodiversity as a central priority. Instead, to ensure that these fragmented ‘islands’ play a fundamental role as ecological patches serving as refugia for many species, the strategy would be to adopt an agriculture centered ‘language’ for nature conservation that calls for the conservation of a complex landscape to safeguard and enhance pollination function within natural habitats and potentially in adjoining agricultural areas. For example, considering pollination, and emphasizing the importance of natural habitats such as pine forests, oak woodlands, and olive groves, as having the greatest overall value for plant pollinator communities and provision of the healthiest pollination services would help establish a link between rural communities and nature [21].

Another recurring theme that emerged from photographs and narratives was the role of family members, in particular grandparents, as having a strong influence on the positive perceptions of nature. This is part of the Lebanese culture where grandparents live in close proximity of their grandchildren and are involved in their upbringing. In our study this is confirmed by the participants’ narratives such as “This is the vine that I used to sit under with my grandfather to rest”, and “This picture demonstrates when I used to come and watch my grandfather plant and mulch”. The following lyrics of a cultural Lebanese song supports this relation with grandparents: “I miss your voice grandmother, I hear your voice coming from far, from the vineyards and the apple trees, your voice holds the sun and has the color of figs and olives and the smell of trees, …” (Fairuz sings "Sitti Ya Sitti" in the Play "Mais Elreem" 1975).

For families that have moved to coastal cities to live and work, grandparents, who remained in villages, are especially important as the guardians of rural living and they help build emotional experiences for youth that lead to positive perceptions of nature and village life. A study by Denham and Smith (1989) [22] has shown the positive influence that grandparents’ social support has on the younger generations especially in relation to family structure and function, socialization, influence patterns, and the transmission of values. Furthermore, Bengtson (2001) [23] suggests that because of increasing longevity and historical changes in family structure, the role and importance of grandparents is increasing overtime. Furthermore, there is rising interest in the role of grandparents and their influence on children’s development and welfare.

Attention is being focused on the positive influence that grandparents’ social support has on the younger generations. Moreover, many publications concerning grandparents address issues such as family structure and function, socialization, influence patterns, and the transmission of values [22]. Furthermore, in an attempt to further elaborate the grandparent/grandchild relationship, a study by Barranti (1985) [24] suggests that as a result of basic demographic changes, this relationship has the potential to span three to four decades of life. In light of this newly emerging family phenomenon, it is suggested that the role of the grandparents as a potential family resource is very essential. Families serve as the genesis and traditional source of values for living in a more environmentally benign, activist and ecologically sustainable manner.

The home is a primary shaper of what children, knowingly and unknowingly, take to school or ‘live’ in the everyday political world of choices [25]. According to Sobel (1996) [26], adults’ involvement in early childhood development influences the youth ability to acquire empathy with the natural world and develop a positive rapport with, and respect for nature. These early years are critical if a positive attitude for nature is to be developed. Other studies show that positive attitude towards nature can be nurtured at a young age though positive encounters with nature [27–30]. On the other hand, children who grow up with little or no regular contact with nature, disconnect their sense of self from the natural world, and are less likely to live in harmony with nature [21,26,30].

## Conclusions

Lebanon is a small mountainous country that is part of the Mediterranean Basin Global Biodiversity Hotspot [31]. The country has taken important steps, in line with the international agenda of the Convention on Biological Diversity, towards biodiversity conservation by establishing nature reserves and protected areas by law and ministerial action [32]. Furthermore, civil society is actively contributing to the increase in environmental awareness and is leading interventions through a relatively large number of registered environmental non-governmental organizations [33]. Nonetheless, these efforts do not seem to halt the deterioration of Lebanon’s natural heritage as rapid urban expansion coupled with political instability is causing environmental degradation and habitat destruction. Unlike coastal ecosystems that have been decimated by towns and cities, the semi-natural mountain agro-ecosystems still constitute a significant component of the country’s natural heritage. The fate of this heritage, however, depends on the extent to which these communities live in harmony with nature. Whether youth in these communities become eco-citizens motivated to care for nature is influenced by the external environment of natural areas and altered habitats, or social mediators such as friends and relatives, teachers, and books. But it is also guided the internal environment of the child’s needs, abilities, emotions, and interests in response to these places and people [27].

The knowledge gained from this project has highly influenced the design of future nature conservation activities by the researchers. We have engaged in the development of informal learning activities that are a reflection of parents and grandparents normal involvement with youth and have revised strategies to engage communities in general in nature conservation focusing in particular on local perspectives of nature, rural lifestyles, agro-pastoral practices, and the environment.
